# Knowledge, Attitude, and Practice (KAP) of COVID-19 Vaccine Among Saudi Mothers

**DOI:** 10.7759/cureus.36826

**Published:** 2023-03-28

**Authors:** Hadeel A Ashour, Sara F Alhinti, Samirah Abdulqader Hawsawi, Arwa A Alsuwailem, Ali AlFarhan, Imad Abdulmajeed

**Affiliations:** 1 College of Medicine, King Saud Bin Abdulaziz University for Health Sciences College of Medicine, Riyadh, SAU; 2 Family Medicine and Primary Health Care, Ministry of the National Guard - Health Affairs, Riyadh, SAU; 3 Family Medicine and Primary Health Care, King Abdulaziz Medical City Riyadh, Riyadh, SAU

**Keywords:** practice, attitude, knowledge, primary clinic, mothers, pandemic, infectious disease, vaccine, coronavirus

## Abstract

Background: The coronavirus disease 2019 (COVID-19) vaccine is a novel vaccine that was created during the midst of the COVID-19 pandemic in 2020 to combat the highly contagious COVID-19 infection. Since the initiation of vaccine administration campaigns globally, lots of research was simultaneously being done to study the vaccine’s side effects and possible complications, especially in vulnerable groups such as pregnant women and children. Saudi Arabia is one of the leading countries in administering the COVID-19 vaccine to its population. However, due to the exchange of a lot of incorrect information through social media platforms about the vaccine’s safety, people, particularly women expecting a child, breastfeeding, or having younger children, started to display some vaccine hesitancy. This study aims to assess the knowledge, attitude, and practice (KAP) of the COVID-19 vaccine among Saudi mothers and to recognize how certain individual characteristics affect it.

Methods: This is an observational cross-sectional study that was carried out among 293 Saudi mothers attending primary healthcare clinics at King Abdulaziz Medical City (KAMC), Riyadh, Saudi Arabia from April 2022 to July 2022. The participants completed a pre-validated self-administered questionnaire that was composed of 39 closed-response questions divided into four sections: participant characteristics, knowledge, attitude, and practice towards the COVID-19 vaccine. The English questionnaire was translated to Arabic, retranslated back to English, and then compared to the first English version by a different translator to ensure translation accuracy. A pilot study was conducted on 20 participants before the survey was distributed for data collection. Statistical analysis was done using SPSS version 23.0 (IBM Corp., Armonk, NY). The association between the four sections of the questionnaire was assessed using Chi-square test of proportion.

Results: The study found that 64% of the participants were below the age of 40. The majority (56%) have earned a bachelor’s or a higher degree. According to 41%, "Ministry of Health official channels" was the most important source of COVID-19 vaccine-related information. Almost half of the respondents (45%) showed to have an excellent knowledge of the COVID-19 vaccine and 62% showed to have a positive attitude towards it. Around 40% of the participants reported that they delayed taking the COVID-19 vaccine until it was mandatory. For those who have children aged between 12 and 18 years, 78% stated that their children took the COVID-19 vaccine. Mothers aged below 40 years showed to have significantly better vaccine knowledge compared to the older group. Mothers who received the influenza vaccine over the past three years were less likely to delay taking the COVID-19 vaccine until it became mandatory compared to those who did not receive it.

Conclusion: Younger age, higher educational level, flu vaccine administration in the previous three years, and adherence to child immunization schedules were all factors that had a significant impact on the KAP towards the COVID-19 vaccine. Correcting misunderstandings about vaccine safety through educational campaigns and providing timely information through the Ministry of Health channels can all contribute to achieving better practice related to vaccine uptake in this group.

## Introduction

The coronavirus disease 2019 (COVID-19), first identified in Wuhan, China, in December 2019, is an infectious disease caused by the severe acute respiratory syndrome coronavirus 2 (SARS-CoV-2) [1]. In March 2020, the World Health Organization (WHO) declared the outbreak of this disease as a pandemic. According to the WHO, building herd immunity with vaccination is so far the best approach to halt the spread of this disease [1]. Saudi Arabia is one of the countries in which the rate of COVID-19 vaccination is high [2].

In December 2020, Saudi Arabia began administering the vaccine to its population, becoming the first Arab country to roll out the Pfizer-BioNTech jab. Up until today, more than 76% of the Saudi population are vaccinated against COVID-19 with at least one dose [3]. These vaccines have been reported to be safe, however, in very rare cases they have been linked with some cases of anaphylaxis and thrombotic incidents [1]. These rare adverse outcomes resulted in some vaccine hesitancy among the Saudi population. Pregnant women displayed greater vaccine hesitancy compared to the general population as little information about the safety of the vaccine among the pregnant population was available at the time [4].

The novelty and speed of creating the COVID-19 vaccine have raised numerous problems that demand a better understanding of the predictors of its acceptance in not only the general population but also vulnerable groups such as women expecting a child or breastfeeding [5]. Social media played an inevitable role in influencing people’s decision to receive the vaccine as scarce information about vaccine safety and side effects was available when the vaccination campaigns began [5-6]. A local study in Saudi Arabia that investigated the knowledge and attitude of the COVID-19 vaccine among the public, pointed out that a big portion of the respondents had satisfactory knowledge and a positive attitude [1]. However, in their study, only 19% of the participants were females. Additionally, they found most participants agreed that the COVID-19 vaccine is not preferred for children above 12 and pregnant women despite the approved national practice of giving the vaccine to these two groups. This encouraged our study to assess the KAP of this vaccine post-pandemic among Saudi mothers.

## Materials and methods

Study setting, population, and sample size

This is an observational cross-sectional study conducted in Riyadh, Saudi Arabia using a self-administered questionnaire. All mothers aged 18-65 years attending primary care clinics at King Abdulaziz Medical City (KAMC), Riyadh during the data collection period were eligible to be part of this study. According to KAMC primary care directorate, the total population served by KAMC primary care clinics is approximately 150,000 patients [2]. Assuming that 50% are women based on a 1:1 ratio from the last population census, our population size is 75,000 [7]. Using the OpenEpi website for sample size calculation, the optimal sample size for this study was calculated to be 280 assuming a 5% margin of error, a 95% confidence level, and a 24% anticipated frequency [1, 8]. A total of 300 Saudi mothers participated in this study. Seven participants provided incompletely filled questionnaires and were excluded from the study leaving a final sample size of 293.

Study instrument

Data were collected from mid-April till mid-July 2022 using a hard copy questionnaire composed of 39 closed-response questions divided into four sections. The first section (questions 1-14) recorded the participants’ socio-demographic data, including age, educational level, and monthly income. The second section (questions 15-24) included knowledge-related statements about the COVID-19 vaccine. In this section, participants were expected to answer with “yes,” “no,” or “I don’t know.” The third section (questions 25-35) assessed respondents’ attitude towards the COVID-19 vaccine using Likert-type scale questions. The last section of the questionnaire included four statements related to the practice of the COVID-19 vaccine. All items included in the questionnaire were adapted from questionnaires used in the previously published KAP studies [1, 9- 11]. The English questionnaire was translated to Arabic, retranslated back to English, and then compared to the first English version by a different translator to ensure translation accuracy. A pilot study was conducted on 20 participants to assess the validity and reliability of the questionnaire. Feedback given by the participants of the pilot study was evaluated by a group of experts and necessary changes were made before the survey was distributed for data collection. 

Ethical clearance

Data collection started only after the study was ethically approved by the Institutional Review Board (IRB) of King Abdullah International Medical Research Center (KAIMRC) on the 10th of February 2022 (reference number: IRB/0602/22). An informed consent sheet was signed by all participants prior to their participation. Participants’ names and other identifying personal data were not used. Instead, each questionnaire was labeled with a serial number to insure the anonymity of all participants.

Scoring criteria

In this study, two scores were calculated, one for knowledge and one for attitude. For the knowledge score, each correct answer carried one point, and each wrong or “I don’t know” answer carried zero points. The maximum knowledge score was ten and the minimum was zero. After calculating the knowledge scores, participants were categorized into three groups: poor knowledge (0 ≤ score ≤ 3), average knowledge (3 < score < 7), and excellent knowledge (score ≥ 7). For the attitude score, “strongly agree” carried five points, “agree” carried four points, “neutral” carried three points, “disagree” carried two points, and “strongly disagree” carried one point. The maximum attitude score was 55 and the minimum was 11. A score of 33 was taken as a cutoff point, participants that scored 33 or less were considered to have a negative attitude, whereas participants that scored more than 33 were considered to have a positive attitude towards the vaccine.

Statistical analysis

Statistical analysis was done using SPSS version 23.0 (IBM Corp., Armonk, NY, USA). All variables in this study are categorical and were described using frequencies and percentages. Crosstabulation with Chi-square test of proportion was applied to assess the association between the respondents’ socio-demographic characteristics and their KAP towards the COVID-19 vaccine. Additionally, crosstabulation with Chi-square test of proportion was also applied to assess the association between the respondents’ knowledge of COVID-19 vaccine and their attitude towards it. In this study, a p-value of less than 0.05 was considered statistically significant.

## Results

 Participants’ characteristics

Most mothers were below the age of 40 (64%, n= 187) and had a bachelor’s degree or higher (56%, n=165). More than half of the participants did not receive the influenza vaccine in the last three years (55%, n=162). Participant’s characteristics are shown in Table 1.

**Table 1:** Participants’ characteristics.

Demographic variable		Frequency (percentage)
Age	Below 40	187 (63.8)
	40 and above	106 (36.2)
Education	No formal education	16 (5.5)
	School education	112 (38.2)
	Bachelor’s degree or higher	165 (56.3)
Occupation	Student	6 (2)
	Unemployed	197 (67.2)
	Employed	83 (28.3)
	Retired	7 (2.4)
Influenza vaccine during the past three years	Vaccinated	131 (44.7)
	Not vaccinated	162 (55.3)
COVID-19 vaccine administration decision	My decision	224 (76.5)
	Influenced by someone	69 (23.5)
COVID-19 vaccination doses	Fully vaccinated	157 (53.6)
	Not fully vaccinated	136 (46.4)
Most important source of COVID-19 vaccine information	Television	49 (16.7)
	Social media and internet	80 (27.3)
	Ministry of health channels	121 (41.3)
	Family and friends	21 (7.2)
	Healthcare providers	22 (7.5)
“Did your children follow their childhood immunization schedule?”	Yes	258 (88.1)
	No	35 (11.9)

COVID-19 vaccine knowledge

Only 45% (n=133) of the respondents had excellent knowledge of the COVID-19 vaccine while the rest had either average or poor knowledge. The most correctly answered knowledge-related question was “Is it true that the administration of the COVID-19 vaccine may cause mild side effects?” 90% (n= 263). The most incorrectly answered knowledge-related question was “Does protective immunity against COVID-19 occur immediately after the first dose?” 66% (n= 195). Table 2 shows the questions of the knowledge section of this study as well as the frequency of response for each question.

**Table 2:** Participants’ knowledge of the COVID-19 vaccine.

Item		Frequency (percentage)
Can you get COVID-19 disease from COVID-19 vaccine?	Yes	38 (13)
	No	121 (41.3)
	I don’t know	134 (45.7)
Can you get COVID-19 infection even after taking COVID-19 vaccine?	Yes	262 (89.4)
	No	6 (2)
	I don’t know	25 (8.5)
Can COVID-19 vaccine be given if you have a past history of COVID-19 infection?	Yes	250 (85.3)
	No	12 (4.1)
	I don’t know	31 (10.6)
Can COVID-19 vaccine be given while you have COVID-19 infection?	Yes	13 (4.4)
	No	213 (72.7)
	I don’t know	67 (22.9)
Can the COVID-19 vaccine be given to pregnant women?	Yes	183 (62.5)
	No	62 (21.2)
	I don’t know	48 (16.4)
Can the COVID-19 vaccine be given to breastfeeding women?	Yes	151 (51.5)
	No	54 (18.4)
	I don’t know	88 (30)
Can the COVID-19 vaccine also protect us from the Influenza virus?	Yes	58 (19.8)
	No	113 (38.6)
	I don’t know	122 (41.6)
Does the immune response from the COVID-19 vaccine go down over time?	Yes	124 (42.3)
	No	37 (12.6)
	I don’t know	132 (45.1)
Does a protective immunity against COVID-19 occur immediately after the first dose?	Yes	97 (33.1)
	No	98 (33.4)
	I don’t know	98 (33.4)
Is it true that the administration of the COVID-19 vaccine may cause mild side effects?	Yes	263 (89.8)
	No	11 (3.8)
	I don’t know	19 (6.5)

Attitude towards the COVID-19 vaccine

**Figure 1:**
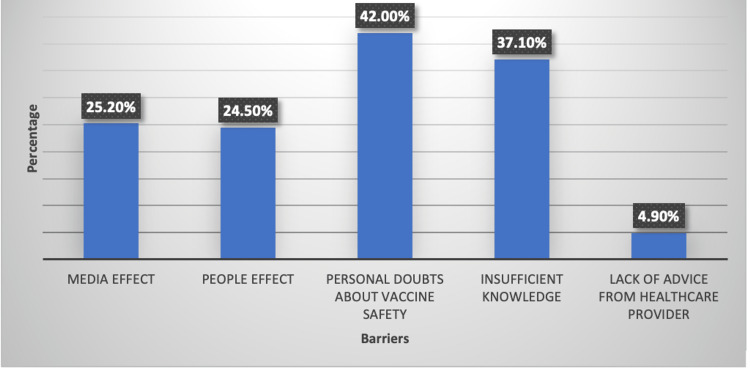
Barriers to receiving a second or third dose of COVID-19 vaccine.

A total of 62% (n=182) of the participants showed a positive attitude towards the COVID-19 vaccine. Yet a big number of participants were still concerned about receiving a second or third dose of the vaccine 44% (n= 129) (Table 3). The most frequently reported barrier to receiving a second or third dose of the vaccine was personal doubt about vaccine safety (42%) (Figure 1).

**Table 3:** Participants’ attitude toward the COVID-19 vaccine.

Item		Frequency (percentage)
COVID-19 vaccines can be harmful to women planning to get pregnant	Strongly agree	41 (14)
	Agree	66 (22.5)
	Neutral	100 (34.1)
	Disagree	74 (25.3)
	Strongly disagree	12 (4.1)
It is important to discuss the risks/benefits of receiving COVID-19 vaccine with my healthcare worker first if I am pregnant, planning to get pregnant, or breastfeeding	Strongly agree	157 (53.6)
	Agree	100 (34.1)
	Neutral	22 (7.5)
	Disagree	10 (3.4)
	Strongly disagree	4 (1.4)
If the next COVID-19 dose was scheduled while I am breastfeeding I will receive it	Strongly agree	41 (14)
	Agree	91 (31.1)
	Neutral	54 (18.4)
	Disagree	62 (21.2)
	Strongly disagree	45 (15.4)
COVID-19 vaccination is being promoted for financial reasons	Strongly agree	25 (8.5)
	Agree	26 (8.9)
	Neutral	79 (27)
	Disagree	108 (36.9)
	Strongly disagree	55 (18.8)
I will advise my family and friends to be vaccinated with the COVID-19 vaccine.	Strongly agree	96 (32.8)
	Agree	106 (36.2)
	Neutral	60 (20.5)
	Disagree	23 (7.8)
	Strongly disagree	8 (2.7)

Practice towards the COVID-19 vaccine

Some 39% (n=115) of the participants reported that they delayed taking the COVID-19 vaccine until it was mandatory by the Ministry of Health (MoH) of Saudi Arabia, and only 10% (n=30) stopped a family member or a friend from taking the vaccine. For those who have children aged between 12 and 18 years, 78% (n=110) stated that their children took the COVID-19 vaccine.

Patient characteristics as predictors of knowledge, attitude, and practice

When comparing various variables together using Chi-square, we found among mothers who were below 40 years of age (47%, n=88) had significantly excellent knowledge (p < 0.001) and (64%, n=119) had a positive attitude (p=0.44). Mothers who had a university or higher education (58%, n=96) had significantly excellent knowledge compared to their peers (p < 0.001). Also, there was a significant relationship between taking the vaccine by a personal decision and having both excellent knowledge (47%, n=106) (p=0.002) and a positive attitude (76%, n=169) (p < 0.001). Being vaccinated with the influenza vaccine (69%, n=90) is significantly related to a positive attitude for the COVID-19 vaccine (p=0.02). Mothers who received the influenza vaccine were also significantly less likely to delay taking the COVID-19 vaccine until it became mandatory (67%, n=88) (p=0.04). In addition, mothers who followed up with their children’s immunization schedule were significantly more likely to have both excellent knowledge (49%, n=127) (p < 0.001) and a positive attitude (67%, n=171) (p < 0.001) towards receiving the COVID-19 vaccine (Table 4). Additionally, they showed significantly better practice in the following areas: First, ‘not delaying the COVID-19 vaccine until it became mandatory’ (64%, n=164) (p=0.007). Second, ‘not stopping a family member or friend from receiving the COVID-19 vaccine’ (93%, n=240) (p < 0.001). Finally, ‘having a child between ages 12 and 18 who received the COVID-19 vaccine’ (85%, n=104) (p < 0.001).

**Table 4:** Participants’ characteristics compared with both knowledge and attitude toward the COVID-19 vaccine. *p values of statistically significant results are tagged with a star.

Variable/Item		Poor knowledge N (%)	Average knowledge N (%)	Excellent knowledge N (%)	p value	Negative attitude N (%)	Positive attitude N (%)	p value
Age	Age below 40	10(5.3)	89(47.6)	88(47.1)	<0.001^*^	67(36)	119(64)	0.44
	Age 40 or above	23(21.7)	38(35.8)	45(42.5)		43(40.6)	63(59.4)	
Education	No formal education	4(25)	9(56.3)	3(18.8)	<0.001^*^	8(50)	8(50)	0.55
	School education	20(17.9)	58(51.8)	34(30.4)		40(36)	71(64)	
	University or higher	9(5.5)	60(36.4)	96(58.2)		62(37.6)	103(62.4)	
COVID-19 vaccination doses	NOT fully vaccinated	22(16.2)	60(44.1)	54(39.7)	0.026^*^	64(47.4)	71(52.6)	0.001^*^
	Fully vaccinated	11(7)	67(42.7)	79(50.3)		46(29.3)	111(70.7)	
COVID-19 vaccine administration decision	Taking the vaccine was my decision	17(7.6)	101 (45.1)	106(47.3)	0.002^*^	54(24.2)	169(75.8)	<0.001^*^
	Taking the vaccine was influenced by family/friends	16(23.2)	26(37.7)	27(39.1)		56(81.2)	13(18.8)	
Influenza vaccine in the last 3 years	Vaccinated	14(10.7)	54(41.2)	63(48.1)	0.7	40(30.8)	90(69.2)	0.02^*^
	Not vaccinated	19(11.7)	73(45.1)	70(43.2)		70(43.2)	92(56.8)	
Adherence to children immunization schedule	No	11(31.4)	18(51.4)	6(17.1)	<0.001^*^	24(68.6)	11(31.4)	<0.001^*^
	Yes	22(8.5)	109(42.2)	127(49.2)		86(33.5)	171(66.5)	

Relationship between knowledge, attitude, and practice towards the COVID-19 vaccine

**Figure 2:**
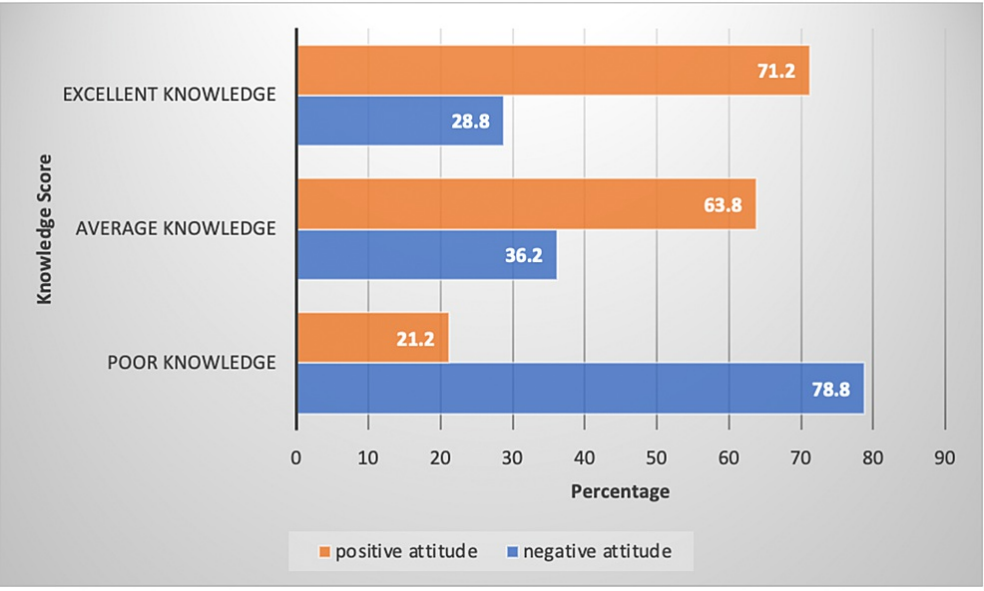
Vaccine knowledge compared to attitude.

 A significant relationship was found between the knowledge of the COVID-19 vaccine and the attitude towards it (p < 0.001). As the knowledge about the COVID-19 vaccine improved, the attitude towards receiving it also improved (Figure 2). Those who had excellent knowledge were significantly less likely to delay taking the vaccine until it became obligatory (68%, n= 91) (p=0.014) (Table 5). Additionally, it was also found that having excellent knowledge was significantly associated with the positive practice of vaccinating children between ages 12 and 18 years with the COVID-19 vaccine (92%, n= 60) (p < 0.001). 

**Table 5:** Knowledge and attitude compared with practice of COVID-19 vaccine. *p values of statistically significant results are tagged with a star.

	“Did you delay taking the vaccine until it became mandatory?”		
	No N (%)	Yes N (%)	p value
Poor knowledge	14(42.4)	19(57.6)	0.014^*^
Average knowledge	73(57.5)	54(42.5)	
Excellent knowledge	91(68.4)	42(31.6)	
Negative attitude	29(26.4)	81(73.6)	<0.001^*^
Positive attitude	148(81.3)	34(18.7)	

## Discussion

COVID-19 was a challenging worldwide pandemic, and ongoing effort is being made by the Saudi MoH to halt the spread of this infection. One of the disease-control and prevention strategies implemented by the Saudi MoH was ensuring public immunization coverage by arranging COVID-19 vaccination programs. The KAP toward a novel vaccine are some factors that might influence the success of such programs [1]. According to our participants, "Ministry of Health Channels" was the most common source of their COVID-19 vaccine-related knowledge. This contradicts a study conducted in Oman that found that social media was the most common source of COVID-19 vaccine-related information among their population [10]. The availability and easy access to validated vaccine-related information from official health channels allow the dissemination of reliable vaccine knowledge to the general public and this is reflected by the good percentage of respondents having “excellent” knowledge of the vaccine in this study.

Despite the fact that getting the vaccine became mandatory in May 2021, (77%) of our participants stated that they received the first dose when they became eligible to receive it based on a personal decision. The level of vaccine acceptance observed in our sample population was higher compared to some other similar studies. The willingness to take the vaccine was found to be (51%) in Russia, (57%) in Oman, and less than (43%) in Egypt [10, 12]. This discrepancy in the levels of vaccine acceptance could possibly indicate that different populations have different levels of COVID-19 vaccine knowledge that influenced the perception towards it.

Results of this study show that most of the participants had either “average” or “excellent” knowledge of the novel vaccine. These findings greatly distinct that of another study conducted in Malaysia in 2021 in which more than half (62%) of the respondents had poor knowledge of the vaccine [11]. This noticeable difference in the level of vaccine knowledge among the two populations might be attributed to the increased vaccine awareness campaigns carried via mass media by 2022 compared to 2021. A common misconception about the vaccine was related to the development of immunity after taking the vaccine. One study conducted in Turkey showed that almost 25% of the participants believe that a protective immunity against COVID-19 occurs immediately after taking the first dose [13]. Similarly, this misconception was seen among 33% of our participants. This highlights the need to put more emphasis on educating the public on how and when effective vaccine immunity starts to develop. 

The majority of mothers had a positive attitude to the COVID-19 vaccine. This contributes to the substantial geographic variation reported by a study done in 16 countries to assess the acceptance of COVID-19 vaccination among pregnant women and mothers [14]. Concerns about the vaccine's safety and efficacy were the main reasons Saudi mothers were reluctant to receive another dose of the vaccine. This is consistent with the findings of Mannan et al. as they reported that lack of confidence was a major predictor of vaccine acceptance [12]. Interestingly, we found that mothers who took the vaccine based on their own personal decision had both excellent knowledge and positive attitude towards the vaccine. This pattern was also observed in a study conducted by Clarke et al. which found that (68%) of pregnant women were not influenced by family or friends when making the decision to be vaccinated [15]. Participants in this study did not rely greatly on information gained from their family and friends, this could be a reflection of their trust in the validity of the information provided by the Ministry of Health. The study found that young mothers who were below 40 years of age showed significantly excellent knowledge and a positive attitude which is in accordance with Al-Zalfawi et al. [1]. We believe the reason younger mothers scored higher in both the knowledge and attitude domains, is because they are more exposed to the internet, thus the online COVID-19-related information was easily accessible to them. Also, they are more likely to be well-educated compared to their older peers. A major finding in the study was that as the knowledge got better, the attitude became more positive, which was also observed in four similar studies [10, 16-18].

Being vaccinated with the influenza vaccine and good adherence to childhood immunizations were significantly associated with a positive attitude and a positive practice of receiving the COVID-19 vaccine. A Singaporean study found similar findings when measuring the association between previous influenza vaccine uptake and attitude towards the COVID-19 vaccine [17]. On the contrary, Mohan et al. found that Qatari perinatal women who were previous acceptors of the influenza vaccine and adherent to childhood immunizations had still shown hesitancy in receiving the COVID-19 vaccine [9]. The Qatari study was done in 2020 and the Singaporean study was conducted a year later, so knowledge about vaccine safety must have dramatically increased and become widely available by then. This great increase in perceived vaccine safety might explain our findings as well. A key finding in the study was that mothers who had excellent knowledge and a positive attitude towards receiving the COVID-19 vaccine were significantly more likely to vaccinate their children with it. Many European countries reported distrust of authorities and doctors as a major reason for refusing a vaccine as opposed to lack of information or misinformation [19]. This is also observed in a Dutch study that found parents’ most common reason for vaccine hesitancy was governmental wariness [20-21]. Since our participants reported "Ministry of Health Channels" to be the most crucial source of vaccine information, this positively reflects how Saudi Arabia’s governmental efforts of providing timely information and building trust with its population had been successful at least among this group of people.

Our study was the first to demonstrate the relationship between knowledge and practice of uptaking the COVID-19 vaccine in Saudi Arabia. We found that higher knowledge scores significantly resulted in better practice of uptaking the vaccine, especially in sensitive domains such as “not delaying vaccine uptake until it became mandatory.” This highlights the importance of the physician's role in continuous patient education. There are some limitations of this study to acknowledge. First, the study was a cross-sectional study with non-randomized sampling. Moreover, the study was conducted in the Riyadh region and might not reflect mothers' knowledge, attitude, and practice across Saudi Arabia. 

## Conclusions

In conclusion, most of the mothers had an overall good KAP of the COVID-19 vaccine. This was significantly associated with younger age, higher educational level, previous influenza vaccine uptake, and adherence to children's immunization. This study proved that knowledge can be a powerful tool in improving vaccine uptake, especially in vulnerable or hesitant groups. These findings should encourage authorities to continuously improve their platforms in providing updated COVID-19 vaccine-related information.
